# Trends and Determinants in Uptake of Cervical Cancer Screening in Spain: An Analysis of National Surveys from 2017 and 2020

**DOI:** 10.3390/cancers14102481

**Published:** 2022-05-18

**Authors:** Silvia Portero de la Cruz, Jesús Cebrino

**Affiliations:** 1 Department of Nursing, Pharmacology and Physiotherapy, Faculty of Medicine and Nursing, University of Córdoba, Avda. Menéndez Pidal, S/N, 14071 Córdoba, Spain; n92pocrs@uco.es; 2 Research Group GC12 Clinical and Epidemiological Research in Primary Care, Instituto Maimónides de Investigación Biomédica de Córdoba (IMIBIC), Hospital Universitario Reina Sofía, 14071 Córdoba, Spain; 3 Department of Preventive Medicine and Public Health, Faculty of Medicine, University of Seville, Avda. Doctor Fedriani, S/N, 41009 Seville, Spain

**Keywords:** cytology, guideline adherence, healthcare disparities, mass screening, uterine cervical neoplasms

## Abstract

**Simple Summary:**

Cervical cancer is the fourth leading cause of death and morbidity in women worldwide. Although screening is a successful technique for lowering these rates, there are still great differences in screening adherence in Spain. The aims of this study were to examine the evolution of cytology screening adherence from 2017 and 2020 and to identify the factors associated with uptake among women in Spain. We found that 73.18% of women had received a cytology test during the previous three years. From 2017 and 2020, there was an increase in cytology screening participation among women aged 45–65 years. Foreigners were less likely to have a cytology test. The characteristics associated with cervical cancer screening that we discovered have significant value for public health initiatives, as they will assist health professionals in identifying women who are less likely to undergo screening and encouraging these women to do so.

**Abstract:**

Cervical cancer rates have declined in industrialized nations as a result of cytology screening programs. However, there are still sizeable differences in screening adherence in Spain. This study aimed to identify the prevalence of cervical cancer screening among women in Spain, to analyze trends in that prevalence from 2017 and 2020 and to identify socio-demographic, health, and lifestyle factors related with adherence to this screening test. We conducted a cross-sectional study of 13,619 women aged 25–65 who participated in the 2017 Spanish National Health Survey and the 2020 European Health Survey for Spain. We used logistic regression to examine the relationship between socio-demographic, health and lifestyle factors and cervical cancer adherence. The prevalence of adherence was 73.18%. Additionally, there was a significant decrease in cervical cancer screening uptake from 2017 and 2020 among women aged 25–44 years (2017: 77.80%, 2020: 75.20%, *p* = 0.02), but an increase in the age group of 45–65 years (2017: 68.93%, 2020: 72.39%, *p* < 0.01) and in foreigners (2017: 64.29%, 2020: 72.29%, *p* < 0.01). Screening for cervical cancer is related with age, educational level, social class, insurance status, visits to the family doctor, alcohol consumption and free time physical exercise.

## 1. Introduction

Cervical cancer is probably the most thoroughly studied and preventable human cancer [1]. However, it remains the fourth most common cancer and the fourth leading cause of cancer mortality in women, with an estimated 604,000 new cases and 342,000 deaths globally in 2020 [2]. In 2020, low- and middle-income countries accounted for over 90% of new cases and deaths worldwide [2]. Furthermore, while the incidence of cervical cancer has declined over time in Western Europe, particularly in Spain [3], which has one of the lowest age-standardized indices [4] partly due to the high coverage of cytological screening programs (72% of Spanish women over the age of 25) [5], the rate has increased in some regions such as Central and Eastern Europe [6] or Africa [7]. These differences in cervical cancer rates are largely due to variations in the prevalence of risk factors between countries, particularly exposure to human papillomavirus (HPV), as well as disparities in the capacity of health care systems to set up early detection programs for cancer lesions [8].

Cervical cancer is largely preventable through both vaccination and screening for precursor lesions, with appropriate follow-up and treatment [9]. At present, there are three vaccines to prevent HPV infection: 9-valent HPV vaccine (Gardasil 9, 9vHPV), 4-valent HPV vaccine (Gardasil, 4vHPV), and 2-valent HPV vaccine (Cervarix, 2vHPV). Each of these vaccines protects against HPV genotypes 16 and 18, which collectively cause about 70% of cervical cancers. Both Gardasil vaccines also protect against HPV genotypes 6 and 11, which cause 90% of genital warts. Gardasil 9 also protects against HPV genotypes 31, 33, 45, 52, and 58 [10]. Despite the three HPV vaccines now available have been shown to reduce the incidence of HPV infection, they do not protect against all HPV genotypes [10]. Moreover, HPV vaccinations have only recently been launched, and unvaccinated elderly women are not immunized against HPV infection [11]. Furthermore, vaccination coverage varies greatly by location and country [12].

Cervical cancer screening includes molecular screening method (HPV DNA testing), visual screening method (visual inspection with acetic acid) and cytology-based screening methods (PAP smear and liquid-based cytology) [13]. HPV DNA testing identifies a group of high-risk carcinogenic HPV genotypes, typically including up to 14 types (HPV16, 18, 31, 33, 35, 39, 45, 51, 52, 56, 58 and 59, which are Group 1 carcinogens, and HPV66 and 68) [9]. Cytology tests (including the Papanicolaou smear test and liquid-based cytology) identify atypical cells on the cervix through the preparation and interpretation of slides using microscopy by a trained expert. Liquid-based cytology requires sophisticated processing to create slides from liquid specimens [14]. Papanicolaou smear test may provide a high number of unsatisfactory slides; however, liquid-based cytologic analysis solves some of these quality difficulties and allows for the performance of both molecular and cytologic tests with a single sample [14]. Visual inspection with acetic acid testing identifies aceto-white lesions that require treatment or additional evaluation by applying dilute acetic acid to the cervix without magnification. This method has been used in resource-constrained settings and nations with limited access to health care. However, the evidence that visual inspection with acetic acid reduces the incidence of cervical cancer is weak [14]. The addition of HPV testing to cervical cytology is one of the most recent improvements to cervical cancer screening standards. HPV-DNA testing can be performed on cervical specimens by signal amplification methods or by nucleic acid amplification with polymerase chain reaction. When high-risk HPV testing is combined with cytology, the sensitivity of a single Papanicolaou test for high-grade neoplasia can be increased from 50–85% to over 100% [9].

Previous research has shown that population-based cancer screening programs outperform opportunistic screenings in terms of reducing overuse and cancer mortality, as well as being more cost-effective and attempting to provide screening to all individuals in the target population, thereby reducing disparities in the access to and uptake of cervical cancer screening [8,15].

Since 2019, the Spanish guidelines for the early detection of cervical cancer recommend cytological screening in women aged 25–34 every three years and HPV testing in women aged 35–65 every five years as part of a population-based screening program [16]. The introduction of the HPV vaccine into the Spanish national immunization program in 2007–2008, HPV detection methods, the first cohorts of women vaccinated against HPV reaching screening age, and advances in scientific knowledge have all prompted a review of the way the Spanish National Health System run the screening program [17]. Unfortunately, a substantial variety in screening strategy persists in Spanish regions, with most programs remaining opportunistic with varying degrees of adherence to national recommendations [18]. These factors, together with the fact that 60.7% of Spanish women diagnosed with cervical cancer have never had any cytology testing [19], are what prompted us to conduct the current study, which aimed to identify the prevalence of cervical cancer screening among women in Spain, to analyze trends in that prevalence from 2017 and 2020 and to identify socio-demographic, health, and lifestyle factors related with adherence to this screening test.

## 2. Materials and Methods

To conduct this cross-sectional study, we used secondary data from the 2017 Spanish National Health Survey (SNHS) [20] and the 2020 European Health Interview Survey for Spain (EHIS) [21]. Both surveys were conducted from October 2016–October 2017 and July 2019–July 2020, respectively, by the National Statistics Institute under the supervision of the Spanish Ministry of Health and Social Affairs. These surveys were carried out by applying home-based personal interviews on a countrywide, representative sample of non-institutionalized subjects aged 15 years and older who had their main family residence in Spain. The team who administered the survey had previously been taught fundamental communication skills, associated processes, and in particular questionnaire training. Before taking the surveys, all the participants completed informed consent forms. More information on the methodology of SNHS 2017 and EHIS 2020 is available elsewhere [22,23].

The following samples of women aged 25–65 were chosen based on the screening guideline age groups [16]: 7695 women in SNHS 2017 and 6914 in EHIS 2020. Despite having similar characteristics to the other women, 990 participants (7.13%) were eventually discarded from the sample because they were unwilling to complete the questionnaires (SNHS 2017: *n* = 441; EHIS 2020: *n* = 549).

**Figure 1:**
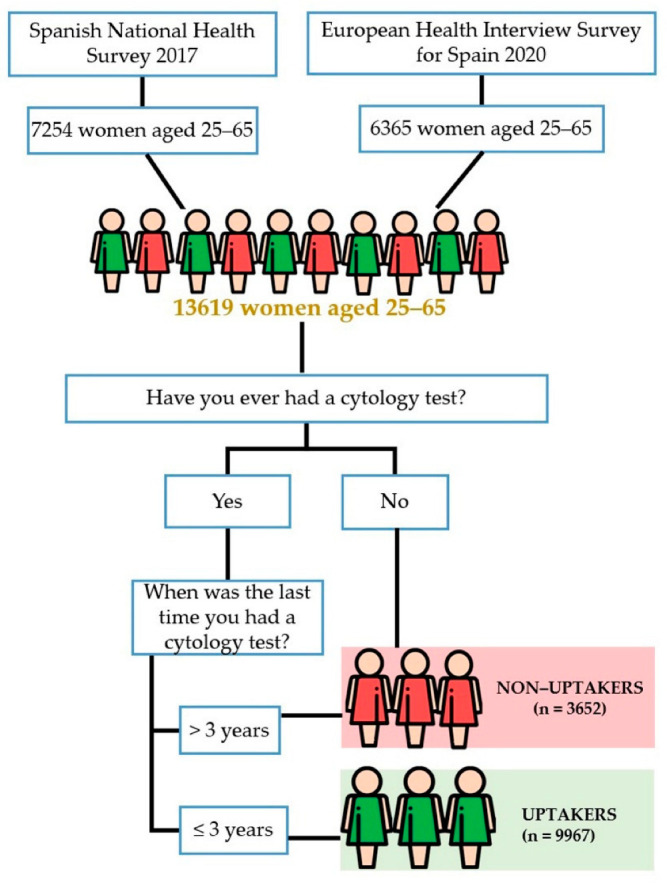
Study workflow.

Our study includes the self-reported responses from these surveys. The variables we used were based on many of the items contained in the questionnaires, which were the same in all the surveys. The dependent variable was cervical cancer screening uptake, which was measured by asking “Have you ever had a cytology test?” Those who answered yes were then asked, “When was the last time you had a cytology test?”. Following the classification of the women who followed the recommended screening period [16], those who admitted to having their most current cytology within the previous three years were referred to as “uptakers”. The other women were referred to as “non-uptakers” (Figure 1).

As independent variables, we examined socio-demographic, health, and lifestyle factors. Age group (25–44, 45–65 years), educational level (without studies, primary, secondary, university), marital status (single, married, widowed, separated or divorced), social class (upper, middle, lower) [24], place of residence (rural/urban) [25] and nationality (Spanish/foreigner) were socio-demographic characteristics. In terms of health, their self-assessed state of health (very good, good, average, bad, very bad), insurance status (public/private) and visits to the family doctor in the preceding four weeks (yes/no) were all considered. The presence of physician-diagnosed mental illnesses, such as chronic anxiety or chronic depression, as well as other psychiatric disorders, was measured by self-reporting. Any woman who was diagnosed with one or more of these three illnesses was termed as “suffering from mental condition”. Finally, body mass index (underweight, normal weight, overweight, obesity) [26], tobacco habit (yes, no), alcohol consumption in the previous year (yes, no) and free time physical exercise (yes, no) were used to measure lifestyle behaviors.

The qualitative variables were reported in terms of frequencies and percentages, while the quantitative variables were expressed in the form of mean and standard deviation (SD). To draw comparisons, the Chi-squared test was applied (from 2017 to 2020, Chi-squared trend analysis was employed widely to discover significant trends in cytology adherence). We also carried out a multivariable analysis to identify which characteristics were independent predictors of cervical cancer screening adherence, and we used the crude and adjusted odds ratio (OR), with their respective 95% confidence intervals, to assess the strength of association. The Hosmer–Lemeshow test was used to evaluate the quality of fit, and to measure the goodness of fit, we examined the adjusted coefficient of determination (R^2^), the F statistic and the normality of the residues. The multivariate model contained only covariates which had a possible association (*p* ≤ 0.15) with the dependent variable, and non-significant variables were discarded using backward selection based on the likelihood of the Wald statistic. The level of statistical significance was set at α = 0.05. SPSS 25.0 software, licensed to the University of Córdoba (Spain), was used to carried out the statistical analysis. The research data are included in the Supplementary File S1.

## 3. Results

**Figure 2:**
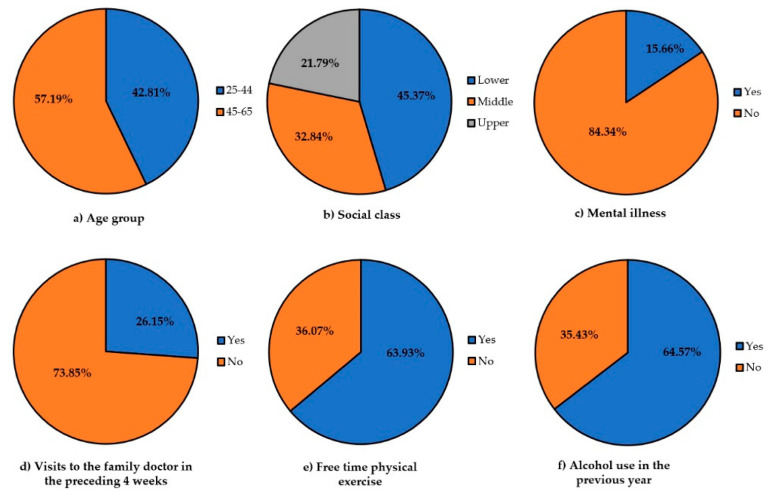
Distribution of socio-demographic factors, health-related characteristics, and lifestyle habits.

We evaluated the data from 13,619 women residing in Spain aged 25 to 65 years old. Most of these women were between the ages of 45 and 65 (57.19%), belonged to the lower socioeconomic class (45.37%) and did free time physical exercise (63.93%) (Figure 2).

Table 1 shows the rates of cervical cytology uptake based on socio-demographic factors, health-related characteristics, and lifestyle habits. A higher uptake rate for cervical cytology was found in the youngest age group, those who were married, with university studies, belonging to the upper social class, of Spanish nationality, without mental illness, with private health insurance, those who visited a general practitioner in the four weeks preceding survey completion, were of normal weight, had consumed alcohol in the previous year and did physical activity during leisure time. 

**Figure 3:**
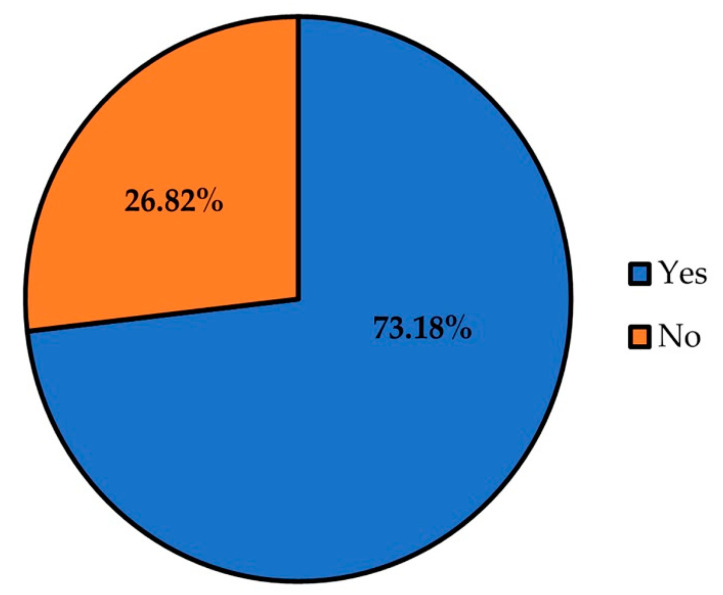
Distribution of adherence to cervical cytology screening of women between 25 and 65 years.

In the previous three years, 73.18% of women between 25 and 65 years had undergone cervical cytology (Figure 3).

There were no variations in cytology testing from 2017 to 2020 (2017: 72.87%, 2020: 73.54%, *p* = 0.38). From 2017 and 2020, we observed an increase in the prevalence of cytology test adherence in foreigners (2017: 64.29%, 2020: 72.29%, *p* < 0.01) (Table 2). 

**Figure 4:**
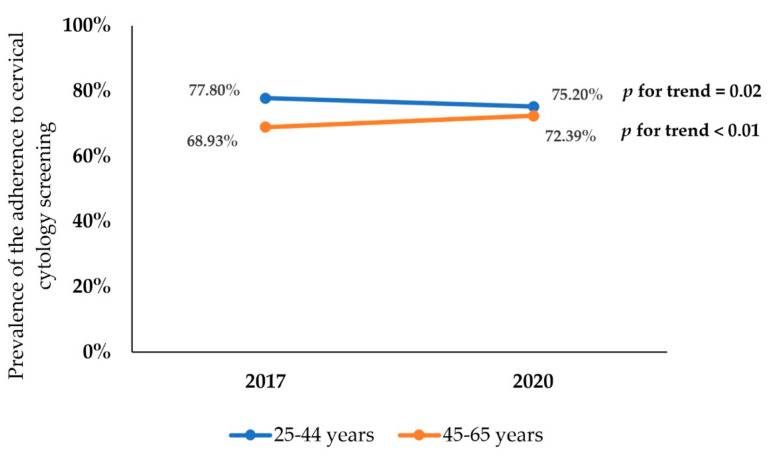
Distribution of adherence to cervical cytology screening according to age group from 2017 to 2020.

Figure 4 illustrates the age-group distribution of cervical cytology screening adherence from 2017 and 2020. Women aged between 45 and 65 years old reported more frequently having undergone cytology screening in 2017 than in 2020 (2017: 68.93%, 2020: 72.39%, *p* < 0.01). However, adherence to cervical cytology decreased across the study years in women aged 25 to 44 (2017: 77.80%, 2020: 75.20%, *p* = 0.02). 

Table 3 shows the crude and adjusted ORs allowing for the identification of determinants of cervical cytology test adherence in the study population. Positive predictors were: age 25–44 (OR = 1.28), educational level (primary: OR = 2.20, secondary OR = 3.41, university OR = 4.28), social class (upper (OR = 1.39), middle (OR = 1.28), private health insurance (OR = 1.67), visits to the primary care physician in the previous four weeks (OR = 1.25), consumption of alcohol in the last year (OR = 1.29) and doing leisure-time physical activity (OR = 1.17). However, foreign nationality was a negative predictor (OR = 0.88). 

## 4. Discussion

### 4.1. Main Findings

The current study analyzed cervical cytology screening adherence in Spain from 2017 and 2020 and shows predictors of cervical cytology testing in a nationally representative sample of women aged 25 to 65. The study also describes the characteristics of women who did not undergo cytology testing. 

In our study, 73.18% of the women surveyed reported undertaking a cervical cytology in the previous three years. This finding is very similar to the cytology uptake value previously reported in Spanish women [27] and may be due to the growing efforts by the Spanish Health System to raise awareness of the benefits of screening for cervical cancer [28]. Moreover, that adherence meets one of the World Health Organization’s stated objectives for 2030 for nations to target the elimination of cervical cancer: 70% of women screened by the age of 35, and again by the age of 45 [29]. Additionally, testing rates vary widely across Europe. The current study’s cervical cytology screening uptake rate lies midway between Northern European nations (ranging from 67% to 94%) and Eastern European countries, which have the lowest prevalence of cytology adherence (10.3%) [30,31]. Nevertheless, in response to the COVID-19 pandemic, screening activities in Spain were reasonably postponed, the priority of periodic cervical cancer screening decreased, and Pap smears were significantly than in March 2019, all of which could have a negative impact on the adherence to cytology screening.

We also looked into potential variables linked to cervical cancer screening uptake, one of the most important of which was age. Our findings revealed that being between the ages of 25 and 44 was related with increased uptake. This is consistent with other findings [32,33], and may be related partly to reproductive years. Younger women may have more frequent gynecologic requirements due to family planning, which may necessitate more frequent examinations and testing, and physicians may be more actively recommending this age group to screen more regularly [34]. Nevertheless, when analyzing the changes from 2017 and 2020, the prevalence of cervical cancer screening adherence differed by age group of women: we observed a reduction in cytology screening uptake among women aged 25–44 and the opposite among those aged 45–65. The decline in screening adherence among younger women over time is especially concerning because women who are screened from an early age receive the most benefit from cervical cancer screening [35]. Here, there is evidence that HPV self-sampling has the potential to increase access to and adoption of screening globally [36], and in fact, self-sampling is now being tested in Spain [37]. Moreover, a number of education techniques, such as phone calls, mailings or group discussion, have been found to be highly successful in increasing cervical cancer compliance [38].

Lower cervical cancer screening rates are linked to a variety of socioeconomic conditions [39]. In general, women with a high level of education and those belonging to higher social status are more likely to undergo cervical cytology [5,40,41,42], which is consistent with the findings from the current study. The link between educational attainment and access to information about cancer screening or the ability to make informed decisions could explain the association between educational level and cervical cancer screening adherence [43], whereas social class is related to the accessibility of cervical cancer screening [44]. These results should be interpreted in the light of the European Union’s problem of overscreening for cervical cancer, which is notably prevalent among women with better socioeconomic level [45].

Across nationality groups, and as previously documented in other research [46,47], our findings demonstrated reduced cervical screening adherence among immigrant women. This is an important public health issue, and a wide range of sociodemographic and psychological factors, such as language difficulties, high mobility, difficult working conditions, and low risk perception, can all contribute to reduced immigrant screening uptake [48,49,50,51]. Surprisingly, our analysis found an increasing trend in the screening of immigrant women from 2017 to 2020. Spain saw an increase in its immigrant population from 2017 and 2020, with the majority coming from South America (2017: 30.64% and 2020: 34.97%) and Morocco (2017: 8.56% and 2020: 10.66%) [52]. Due to a concurrent expansion in population-based cervical screening programs in the different autonomous communities of Spain in those research years, immigration may have played a role in the observed increase in cervical cancer screening uptake over time [18]. Additionally, immigrant-specific cervical cancer screening interventions have been implemented in recent years to motivate this population to participate in screening programs, including ongoing dialogues with the family and community support, removing the social stigma of cervical cancer screening, improving women’s awareness and ability to navigate the health care system (e.g., by translating screening messages into more familiar languages) or providing transportation [18]. We were unable to shed more light on the characteristics of lower adherent migrants due to a lack of data on their migration (time since migration or place of birth). Since there are few studies on the factors which promote and impede participation in cervical cancer screening among migrant women in Spain, further work is needed in this area.

Health insurance coverage is a significant predictor of participation in cancer screening [53]. According to our findings, women with supplementary private health insurance reported more frequently that they had had a screening in the last three years. This extra insurance provides access to specialized and preventative health care services with shorter waiting lists than public services, which contributes to increased uptake [54]. Furthermore, it is possible that having additional private insurance is related to other factors that may influence screening uptake, such as belonging to a privileged social class, the value placed on health or the insured person’s knowledge of the health system [55], all of which increase the expected value of a screening test. Although the results in our study linking having supplementary insurance and higher compliance with cervical cancer screening are supported by the literature [56], some studies showed no such link [42,57]. It is particularly interesting to note that earlier studies have also shown that women with private insurance are more likely to report that they have not undergone screening [33,58]. This disparity might be explained by the fact that women with private insurance do not have a referral from a health care professional.

As previously described by other authors [42], frequent visits to the general practitioner positively impacted adherence to cervical cytology in the current research. As general practitioners provide health services that are closest to the community, they are respected, trusted members of the local community and may play an important role in health communication, such as mobilizing people to take part in screening programs [59].

In terms of lifestyle habits, women residing in Spain were shown to be more likely to undergo screening for cervical cancer if they consumed alcohol and engaged in physical activity during their free time. In Spain, moderate alcohol use is common among women with a high socioeconomic status, which is linked to greater utilization of cancer screening [27]. Although our results are in keeping with earlier research [35,60], data on the effect of alcohol use on cervical cancer screening adherence are conflicting. Some authors found no association [61,62], while others discovered a negative association [27]. According to these findings, further studies are needed to clarify the association between alcohol use and screening participation. Regarding physical activity, studies conducted in Lithuania and Spain found a beneficial effect on screening uptake [27,35]. It was expected that women who engaged in healthy activities would be more health conscious than those who engaged in unhealthy behaviors, and hence more likely to participate in cancer screening [63]. It is noteworthy that contradictory findings on this association have been reported, such as high rates of screening uptake among women who do little physical activity [64], implying that primary prevention programs should target all the populations, including those with healthy habits, rather than just those who lead more unhealthy lifestyles.

### 4.2. Strengths and Limitations

One of the major strengths of this study is the use of a large, nationally representative sample of women, which contributes to the generalizability of the findings. Nevertheless, there are also a number of study limitations. First, since this study has a cross-sectional design, the causality of associations cannot be determined. The second limitation is the information and social desirability bias, which may lead to an overestimation of cervical cancer screening adherence. Finally, because of the varied sources of uptake data, as well as the coexistence of different types of screening programs in different countries and their target populations, comparisons should be made with caution.

### 4.3. Implications for Research and Practice

The World Health Organization strategy for cervical cancer elimination suggests that each country should meet the 90-70-90 targets by 2030 (coverage of 90% of girls vaccinated, 70% of women screened, and treatment of 90% of women identified with cervical disease). Nevertheless, obtaining and maintaining the second target would be one of the most significant issues for many low- and middle-income countries [65]. Findings of our study may be useful in achieving that target recognizing and addressing the factors that are likely to influence women’s disposition towards cervical cancer screening in order to explore focused interventions as effective ways to promote cervical cancer screening adherence. In high-income countries where some of these targets have been met, previous studies have shown that improvements in test performance and screening coverage have a greater effect on elimination timing than increases in vaccination coverage [66,67]. The results of the current study revealed population categories with low screening adherence that may be appropriate target groups for intervention to encourage screening behaviors for detecting cervical cancer in these countries.

On the other hand, health providers must be aware of the sectors of the population at high risk of non-participation and urge screening among these women. Moreover, communication skills, as well as the ability to gather information, are required to raise women’s awareness of the etiology of cervical cancer, HPV infection, preventative strategies, and early detection. It would also be desirable to analyze the influence of the HPV vaccination on uptake of cervical cancer screening programs, as well as the efficiency of the vaccine in decreasing cervical cancer incidence and mortality, when further years of follow-up data from the cohort of immunized Spanish women become available.

## 5. Conclusions

In Spain, the adherence rate for cervical cancer screening is 73.18%. Despite the fact that adherence has increased in recent years among women aged 45–65 and foreigners, discrepancies based on many socio-demographic, health, and lifestyle variables remain. Age between 25 and 44, higher educational level, higher social status, private health insurance, frequent visits to the general practitioner, alcohol use, and physical activity are all positive predictors of cervical screening uptake, but immigration is a negative predictor.

## Figures and Tables

**Table 1:** Uptake of cervical cytology according to socio-demographic, health, and lifestyle variables (n = 13,619).

Variables	Cervical Cytology			
	Total n (%)	Yes n = 9967 (%)	No n = 3652 (%)	*p*-Value
Age group				<0.001
45–65 years old	7789 (57.19)	5499 (70.60)	2290 (29.40)	
25–44 years old	5830 (42.81)	4468 (76.64)	1362 (23.36)	
Educational level				<0.001
Without studies	57 (0.42)	21 (36.84)	36 (63.16)	
Primary	1877 (13.78)	1118 (59.56)	759 (40.44)	
Secondary	7762 (56.99)	5653 (72.83)	2109 (27.17)	
University	3923 (28.81)	3175 (80.93)	748 (19.07)	
Marital status				<0.001
Single	3235 (23.75)	2234 (69.06)	1001 (30.94)	
Married	8134 (59.73)	6158 (75.71)	1976 (24.29)	
Widowed	588 (4.32)	348 (59.18)	240 (40.82)	
Separated or divorced	1662 (12.20)	1227 (73.83)	435 (26.17)	
Social class				<0.01
Lower	6179 (45.37)	4178 (67.62)	2001 (32.38)	
Middle	4473 (32.84)	3390 (75.79)	1083 (24.21)	
Upper	2967 (21.79)	2399 (80.86)	568 (19.14)	
Place of residence				0.79
Urban	4336 (31.84)	3167 (73.04)	1169 (26.96)	
Rural	9283 (68.16)	6800 (73.25)	2483 (26.75)	
Nationality				<0.001
Spanish	12,158 (89.27)	8959 (73.69)	3199 (26.31)	
Foreigner	1461 (10.73)	1008 (68.99)	453 (31.01)	
Mental illness				<0.01
No	11,486 (84.34)	8455 (73.61)	3031 (26.39)	
Yes	2133 (15.66)	1512 (70.89)	621 (29.11)	
Self-assessed state of health				0.14
Very good	2982 (21.90)	2194 (73.57)	788 (26.43)	
Good	7095 (52.10)	5235 (73.78)	1860 (26.22)	
Average	2648 (19.44)	1910 (72.13)	738 (27.87)	
Bad	685 (5.03)	481 (70.22)	204 (29.78)	
Very bad	209 (1.53)	147 (70.33)	62 (29.67)	
Insurance status				
Public	12,981 (95.32)	6422 (72.58)	3559 (27.42)	<0.001
Private	638 (4.68)	545 (85.42)	93 (14.58)	
Visits to the family doctor in the preceding 4 weeks				
No	10,058 (73.85)	7310 (72.68)	2748 (27.32)	0.03
Yes	3561 (26.15)	2657 (74.61)	904 (25.39)	
Body Mass Index				<0.001
Normal weight	73.27 (53.80)	5581 (76.17)	1746 (23.83)	
Underweight	417 (3.06)	317 (76.02)	100 (23.98)	
Overweight	3915 (28.75)	2760 (70.50)	1155 (29.50)	
Obesity	1960 (14.39)	1309 (66.79)	651 (33.21)	
Tobacco habit				0.28
No	9985 (73.32)	7332 (73.43)	2653 (26.57)	
Yes	3634 (26.68)	2635 (72.51)	999 (27.49)	
Alcohol use in the previous year				<0.001
No	4825 (35.43)	3266 (67.69)	1559 (32.31)	
Yes	8794 (64.57)	6701 (76.20)	2093 (23.80)	
Free time physical exercise				<0.001
No	4912 (36.07)	3411 (69.44)	1501 (30.56)	
Yes	8707 (63.93)	6556 (75.30)	2151 (24.70)	

**Table 2:** Distribution of cervical cytology screening adherence according to the study variables from 2017 and 2020 (n = 9967).

Variables	Cervical Cytology (n = 9967)		
	2017 n = 5286 (%)	2020 n = 4681 (%)	*p*-Value
Educational level			
Without studies	13 (38.24)	8 (34.78)	0.79
Primary	624 (57.88)	494 (61.83)	0.08
Secondary	3030 (73.05)	2623 (72.58)	0.64
University	1619 (81.19)	1556 (80.66)	0.67
Marital status			
Single	1152 (69.95)	1082 (68.14)	0.27
Married	3330 (75.10)	2828 (76.43)	0.16
Widowed	184 (57.50)	164 (61.19)	0.28
Separated or divorced	620 (72.68)	607 (75.03)	0.38
Social class			
Lower	2283 (67.11)	1895 (68.24)	0.38
Middle	1765 (75.82)	1625 (75.76)	0.97
Upper	1238 (81.23)	1161 (80.46)	0.34
Place of residence			
Urban	1643 (71.43)	1524 (74.85)	0.10
Rural	3643 (73.54)	3157 (72.93)	0.51
Nationality			
Spanish	4899 (73.62)	4060 (73.74)	0.91
Foreigner	387 (64.29)	621 (72.29)	<0.01
Mental illness			
No	4432 (73.76)	4023 (73.45)	0.71
Yes	854 (68.59)	658 (74.10)	0.18
Self-assessed state of health			
Very good	1090 (74.25)	1104 (72.92)	0.41
Good	2740 (73.72)	2495 (73.86)	0.89
Average	1094 (71.04)	896 (73.65)	0.14
Bad	271 (68.81)	210 (72.41)	0.28
Very bad	91 (67.91)	56 (74.67)	0.38
Insurance status			
Public	5002 (72.29)	4420 (72.91)	0.43
Private	284 (84.78)	261 (86.14)	0.63
Visits to the family doctor in the preceding 4 weeks			
No	3721 (72.72)	3589 (72.64)	0.93
Yes	1565 (73.23)	1092 (76.69)	0.25
Body Mass Index			
Normal weight	2974 (76.63)	2607 (75.65)	0.33
Underweight	160 (71.43)	157 (81.35)	0.12
Overweight	1433 (69.36)	1327 (71.77)	0.09
Obesity	719 (66.39)	590 (67.27)	0.68
Tobacco habit			
No	3826 (73.38)	3506 (73.49)	0.90
Yes	1460 (71.57)	1175 (73.71)	0.15
Alcohol use in the previous year			
No	1773 (67.75)	1493 (67.62)	0.92
Yes	3513 (7516)	3188 (76.69)	0.31
Free time physical exercise			
No	1856 (69.95)	1555 (70.05)	0.41
Yes	3430 (75.19)	3126 (75.42)	0.80

**Table 3:** Variables associated with uptake of cervical cytology among women residing in Spain (2017–2020).

Variables	OR (CI 95%)	OR ^a^ (CI 95%)	*p*-Value
Age group			
45–65 years old	Reference	Reference	
25–44 years old	1.37 (1.26–1.48)	1.28 (1.18–1.39)	<0.001
Educational level			
Without studies	Reference	Reference	
Primary	2.53 (1.46–4.36)	2.20 (1.27–3.82)	<0.001
Secondary	4.60 (2.68–7.89)	3.41 (1.98–5.89)	<0.01
University	7.28 (4.22–12.54)	4.28 (2.46–7.45)	<0.001
Marital status			
Single	Reference		
Married	1.40 (1.28–1.53)		
Widowed	0.65 (0.54–0.78)		
Separated or divorced	1.26 (1.11–1.44)		
Social class			
Lower	Reference	Reference	
Middle	1.50 (1.38–1.64)	1.28 (1.17–1.40)	<0.001
Upper	2.02 (1.82–2.25)	1.39 (1.22–1.58)	<0.001
Place of residence			
Urban	Reference		
Rural	1.01 (0.93–1.10)		
Nationality			
Spanish	Reference	Reference	
Foreigner	0.76 (0.71–0.89)	0.88 (0.78–0.95)	0.04
Mental illness			
No	Reference		
Yes	0.87 (0.79–0.97)		
Self-assessed state of health			
Very good	Reference		
Good	1.01 (0.92–1.11)		
Average	0.93 (0.83–1.05)		
Bad	0.85 (0.71–1.01)		
Very bad	0.85 (0.63–1.16)		
Insurance status			
Public	Reference	Reference	
Private	2.21 (1.77–2.78)	1.67 (1.33–2.10)	<0.001
Visits to the family doctor in the preceding 4 weeks			
No	Reference	Reference	
Yes	1.11 (1.01–1.21)	1.25 (1.14–1.37)	<0.001
Body Mass Index			
Normal weight	Reference		
Underweight	0.99 (0.79–1.25)		
Overweight	0.75 (0.69–0.82)		
Obesity	0.63 (0.57–0.70)		
Tobacco habit			
No	Reference		
Yes	0.96 (0.88–1.04)		
Alcohol use in the previous year			
No	Reference	Reference	
Yes	1.53 (1.41–1.65)	1.29 (1.19–1.40)	<0.001
Free time physical exercise			
No	Reference	Reference	
Yes	1.34 (1.24–1.45)	1.17 (1.08–1.27)	<0.001

OR, odds ratio; OR ^a^, odds ratio adjusted for all sociodemographic, health and lifestyle variables; CI 95%, 95% Confidence Interval. Hosmer–Lemeshow test χ^2^ = 5.80, *p* = 0.15; Nagelkerke’s R^2^ Square = 0.53; *p*-value < 0.01.

## Data Availability

The data presented in this study are available as Supplementary Material (File S1: Research data).

## Supplementary Materials

The following are available online at https://www.mdpi.com/article/10.3390/cancers14102481/s1, File S1: Research data.

## References

[1] Wentzensen N., Schiffman M. (2018). Accelerating cervical cancer control and prevention. Lancet Public Health.

[2] Sung H., Ferlay J., Siegel R.L., Laversanne M., Soerjomataram I., Jemal A., Bray F. (2021). Global Cancer Statistics 2020: GLOBOCAN Estimates of incidence and mortality worldwide for 36 cancers in 185 Countries. CA Cancer J. Clin..

[3] Lin S., Gao K., Gu S., You L., Qian S., Tang M., Wang J., Chen K., Jin M. (2021). Worldwide Trends in Cervical Cancer Incidence and Mortality, with Predictions for the next 15 Years. Cancer.

[4] He W.-Q., Li C. (2021). Recent Global Burden of Cervical Cancer Incidence and Mortality, Predictors, and Temporal Trends. Gynecol. Oncol..

[5] Cobo-Cuenca A.I., Rodríguez-Borrego M.-A., Hidalgo-Lópezosa P., Rodríguez-Muñoz P.M., Martins M., Carmona-Torres J.M. (2018). Prevalence and determinants in cytology testing for cervical cancer screening in Spain (2006-14). Eur. J. Public Health.

[6] Small W., Peltecu G., Puiu A., Corha A., Cocîrṭă E., Cigăran R.G., Plante M., Jhingran A., Stang K., Gaffney D. (2021). Cervical cancer in Eastern Europe: Review and proceedings from the cervical cancer research conference. Int. J. Gynecol. Cancer.

[7] Mahantshetty U., Lavanya G., Grover S., Akinfenwa C.A., Carvalho H., Amornwichet N. (2021). Incidence, treatment and outcomes of cervical cancer in low- and middle-income countries. Clin. Oncol..

[8] De Prez V., Jolidon V., Willems B., Cullati S., Burton-Jeangros C., Bracke P. (2021). Cervical cancer screening programs and their context-dependent effect on inequalities in screening uptake: A dynamic interplay between public health policy and welfare state redistribution. Int. J. Equity Health.

[9] Bedell S.L., Goldstein L.S., Goldstein A.R., Goldstein A.T. (2020). Cervical cancer screening: Past, present, and future. Sex. Med. Rev..

[10] World Health Organization (2017). Human Papillomavirus Vaccines: WHO Position Paper, May 2017. Wkly. Epidemiol. Rec..

[11] Gallagher K.E., LaMontagne D.S., Watson-Jones D. (2018). Status of HPV vaccine introduction and barriers to country uptake. Vaccine.

[12] Altobelli E., Rapacchietta L., Profeta V.F., Fagnano R. (2019). HPV-Vaccination and Cancer Cervical Screening in 53 WHO European Countries: An Update on Prevention Programs According to Income Level. Cancer Med..

[13] World Health Organization (2021). WHO Guideline for Screening and Treatment of Cervical Pre-Cancer Lesions for Cervical Cancer Prevention.

[14] Bouvard V., Wentzensen N., Mackie A., Berkhof J., Brotherton J., Giorgi-Rossi P., Kupets R., Smith R., Arrossi S., Bendahhou K. (2021). The IARC Perspective on Cervical Cancer Screening. N. Engl. J. Med..

[15] Jansen E.E.L., Zielonke N., Gini A., Anttila A., Segnan N., Vokó Z., Ivanuš U., McKee M., de Koning H.J., de Kok I.M.C.M. (2020). Effect of organised cervical cancer screening on cervical cancer mortality in Europe: A systematic review. Eur. J. Cancer.

[16] Ministry of Health, Consumer Affairs and Social Welfare (2022). Cribado Poblacional—Cancer Cervical.

[17] Ministerio de Sanidad, Consumo y Bienestar Social (2019). Orden SCB/480/2019, de 26 de Abril, por la Que se Modifican los Anexos I, III y VI del Real Decreto 1030/2006, de 15 de Septiembre, por el que se Establece la Cartera de Servicios Comunes del Sistema Nacional de Salud y el Procedimiento Para su Actualización.

[18] Molina A., Moreno J., Peiró R., Arroyo G., Ibáñez J., Vanaclocha M., Binefa G., García M., Salas D. (2021). Inequalities in access to cancer screening programmes in Spain and how to reduce them: Data from 2013 and 2020. Rev. Esp. Salud Publica.

[19] Castillo M., Astudillo A., Clavero O., Velasco J., Ibáñez R., de Sanjosé S. (2016). Poor cervical cancer screening attendance and false negatives. A call for organized screening. PLoS ONE.

[20] Ministry of Health, Consumer Affairs and Social Welfare, National Institute of Statistics (2017). Spanish National Health Survey 2017.

[21] Ministry of Health, Consumer Affairs and Social Welfare, National Institute of Statistics (2020). European Health Interview Survey for Spain.

[22] Ministry of Health, Consumer Affairs and Social Welfare, National Institute of Statistics (2017). Spanish National Health Survey 2017: Methodology.

[23] Ministry of Health, Consumer Affairs and Social Welfare, National Institute of Statistics (2020). European Health Interview Survey for Spain 2020: Methodology.

[24] Domingo-Salvany A., Bacigalupe A., Carrasco J.M., Espelt A., Ferrando J., Borrell C. (2013). Proposals for social class classification based on the Spanish National Classification of Occupations 2011 using neo-Weberian and neo-Marxist approaches 2011. Gac. Sanit..

[25] International Labour Office (2018). Rural-Urban Labour Statistics.

[26] World Health Organization (2021). Body Mass Index (BMI). http://www.euro.who.int/en/health-topics/disease-prevention/nutrition/a-healthy-lifestyle/body-mass-index-bmi.

[27] Zamorano-Leon J.J., López-de-Andres A., Álvarez-González A., Astasio-Arbiza P., López-Farré A.J., de-Miguel-Diez J., Jiménez-García R. (2020). Reduction from 2011 to 2017 in adherence to breast cancer screening and non-improvement in the uptake of cervical cancer screening among women living in Spain. Maturitas.

[28] Spanish Society of Medical Oncology Cervical Cancer. https://seom.org/info-sobre-el-cancer/cervix.

[29] World Health Organization (2020). Global Strategy to Accelerate the Elimination of Cervical Cancer as a Public Health Problem.

[30] Williams J., Rakovac I., Victoria J., Tatarinova T., Corbex M., Barr B., Rose T., Sturua L., Obreja G., Andreasyan D. (2021). Cervical cancer testing among women aged 30-49 years in the WHO European Region. Eur. J. Public Health.

[31] International Agency for Research on Cancer (2017). Cancer Screening in the European Union (2017). Report on the Implementation of the Council Recommendation on Cancer Screening.

[32] Tavasoli S.M., Kane E., Chiarelli A.M., Kupets R. (2018). Women’s behaviors toward mammogram and Pap Test: Opportunities to increase cervical cancer screening participation rates among older women. Women's Health Issues.

[33] Johnson N.L., Head K.J., Scott S.F., Zimet G.D. (2020). Persistent disparities in cervical cancer screening uptake: Knowledge and sociodemographic determinants of Papanicolaou and Human Papillomavirus testing among women in the United States. Public Health Rep..

[34] Rendle K.A., Schiffman M., Cheung L.C., Kinney W.K., Fetterman B., Poitras N.E., Lorey T., Castle P.E. (2018). Adherence patterns to extended cervical screening intervals in women undergoing Human Papillomavirus (HPV) and cytology cotesting. Prev. Med..

[35] Petkeviciene J., Ivanauskiene R., Klumbiene J. (2018). Sociodemographic and lifestyle determinants of non-attendance for cervical cancer screening in Lithuania, 2006–2014. Public Health.

[36] Nishimura H., Yeh P.T., Oguntade H., Kennedy C.E., Narasimhan M. (2021). HPV Self-sampling for cervical cancer screening: A systematic review of values and preferences. BMJ Glob. Health.

[37] Serrano B., Ibáñez R., Robles C., Peremiquel-Trillas P., de Sanjosé S., Bruni L. (2022). Worldwide use of HPV self-sampling for cervical cancer screening. Prev. Med..

[38] Agide F.D., Garmaroudi G., Sadeghi R., Shakibazadeh E., Yaseri M., Koricha Z.B., Tigabu B.M. (2018). A systematic review of the effectiveness of health education interventions to increase cervical cancer screening uptake. Eur. J. Public Health.

[39] Murfin J., Irvine F., Meechan-Rogers R., Swift A. (2020). Education, income and occupation and their influence on the uptake of cervical cancer prevention strategies: A systematic review. J. Clin. Nurs..

[40] Willems B., Bracke P. (2018). The education gradient in cancer screening participation: A consistent phenomenon across Europe?. Int. J. Public Health.

[41] Ayenew A.A., Zewdu B.F., Nigussie A.A. (2020). Uptake of cervical cancer screening service and associated factors among age-eligible women in Ethiopia: Systematic review and meta-analysis. Infect. Agents Cancer.

[42] Nunes M.F., Leite A.H., Dias S.F. (2021). Inequalities in adherence to cervical cancer screening in Portugal. Eur. J. Cancer Prev..

[43] Willems B., Cullati S., Prez V.D., Jolidon V., Burton-Jeangros C., Bracke P. (2020). Cancer screening participation and gender stratification in Europe. J. Health Soc. Behav..

[44] Bao H., Zhang L., Wang L., Zhang M., Zhao Z., Fang L., Cong S., Zhou M., Wang L. (2018). Significant variations in the cervical cancer screening rate in china by individual-level and geographical measures of socioeconomic status: A multilevel model analysis of a nationally representative survey dataset. Cancer Med..

[45] Alber J.M., Brewer N.T., Melvin C., Yackle A., Smith J.S., Ko L.K., Crawford A., Glanz K. (2018). Reducing overuse of cervical cancer screening: A systematic review. Prev. Med..

[46] Gallo F., Caprioglio A., Castagno R., Ronco G., Segnan N., Giordano L. (2017). Inequalities in cervical cancer screening utilisation and results: A comparison between italian natives and immigrants from disadvantaged countries. Health Policy.

[47] Barrera-Castillo M., Fernández-Peña R., Del Valle-Gómez M.D.O., Fernández-Feito A., Lana A. (2020). Social integration and gynecologic cancer screening of immigrant women in Spain. Gac. Sanit..

[48] Bucchi D., Chiavarini M., Bianconi F., Galeotti M.E., Gili A., Stracci F. (2019). Immigration, screening, and cervical cancer incidence: An application of age-period-cohort analysis. Eur. J. Cancer Prev..

[49] Ferdous M., Lee S., Goopy S., Yang H., Rumana N., Abedin T., Turin T.C. (2018). Barriers to cervical cancer screening faced by immigrant women in Canada: A systematic scoping review. BMC Women’s Health.

[50] Jang S.H., Meischke H., Ko L.K. (2021). The impact of medical tourism on cervical cancer screening among immigrant women in the U.S. BMC Women’s Health.

[51] Alam Z., Shafiee Hanjani L., Dean J., Janda M. (2021). Cervical cancer screening among immigrant women residing in Australia: A systematic review. Asia. Pac. J. Public Health.

[52] National Institute of Statistics Flow of Immigration from Abroad by Year, Country of Origin and Nationality. https://www.ine.es/jaxiT3/Datos.htm?t=24295#!tabs-grafico.

[53] Zhao G., Okoro C.A., Li J., Town M. (2018). Health insurance status and clinical cancer screenings among US adults. Am. J. Prev. Med..

[54] Garrido-Cumbrera M., Borrell C., Palència L., Espelt A., Rodríguez-Sanz M., Pasarín M.I., Kunst A. (2010). Social class inequalities in the utilization of health care and preventive services in Spain, a country with a National Health System. Int. J. Health Serv..

[55] Walsh B., Silles M., O’Neill C. (2012). The role of private medical insurance in socio-economic inequalities in cancer screening uptake in Ireland: Private insurance and medical screening in the Republic of Ireland. Health Econ..

[56] Fuzzell L.N., Perkins R.B., Christy S.M., Lake P.W., Vadaparampil S.T. (2021). Cervical cancer screening in the United States: Challenges and potential solutions for underscreened groups. Prev. Med..

[57] Freund K.M., Reisinger S.A., LeClair A.M., Yoon G.H., Al-Najar S.M., Young G.S., González E.T., Oliveri J.M., Paskett E.D. (2019). Insurance stability and cancer screening behaviors. Health Equity.

[58] Suk R., Hong Y.-R., Rajan S.S., Xie Z., Zhu Y., Spencer J.C. (2022). Assessment of US preventive services task force guideline-concordant cervical cancer screening rates and reasons for underscreening by age, race and ethnicity, sexual orientation, rurality, and insurance, 2005 to 2019. JAMA Netw. Open.

[59] Gyulai A., Nagy A., Pataki V., Tonté D., Ádány R., Vokó Z. (2018). General practitioners can increase participation in cervical cancer screening—A model program in Hungary. BMC Fam. Pract..

[60] Harder E., Hertzum-Larsen R., Frederiksen K., Kjær S.K., Thomsen L.T. (2020). Non-participation in cervical cancer screening according to health, lifestyle and sexual behavior: A Population-Based Study of nearly 15,000 Danish women aged 23–45 years. Prev. Med..

[61] Pelullo C.P., Cantore F., Lisciotto A., Di Giuseppe G., Pavia M. (2021). Organized breast and cervical cancer screening: Attendance and determinants in southern Italy. Cancers.

[62] Issa T., Babi A., Azizan A., Alibekova R., Khan S.A., Issanov A., Chan C.K., Aimagambetova G. (2021). Factors associated with cervical cancer screening behaviour of women attending gynaecological clinics in Kazakhstan: A Cross-Sectional study. Women’s Health.

[63] Venturelli F., Sampaolo L., Carrozzi G., Zappa M., Giorgi Rossi P. (2019). Associations between cervical, breast and colorectal cancer screening uptake, chronic diseases and health-related behaviours: Data from the Italian PASSI Nationwide Surveillance. Prev. Med..

[64] Ng’ang’a A., Nyangasi M., Nkonge N.G., Gathitu E., Kibachio J., Gichangi P., Wamai R.G., Kyobutungi C. (2018). predictors of cervical cancer screening among Kenyan women: Results of a nested case-control study in a nationally representative survey. BMC Public Health.

[65] Pham M.-A., Benkortbi K., Kenfack B., Tincho Foguem E., Sormani J., Wisniak A., Lemoupa Makajio S., Manga E., Vassilakos P., Petignat P. (2022). Recruitment strategies to promote uptake of cervical cancer screening in the West Region of Cameroon. BMC Public Health.

[66] Burger E.A., Smith M.A., Killen J., Sy S., Simms K.T., Canfell K., Kim J.J. (2020). projected time to elimination of cervical cancer in the USA: A Comparative Modelling Study. Lancet Public Health.

[67] Hall M.T., Simms K.T., Lew J.-B., Smith M.A., Brotherton J.M., Saville M., Frazer I.H., Canfell K. (2019). the projected timeframe until cervical cancer elimination in Australia: A modelling study. Lancet Public Health.

